# “And Then He Hit Me.” Disclosure Patterns in Forensic Interviews of Preschool-Aged Allegedly Abused Children

**DOI:** 10.1177/10775595251328884

**Published:** 2025-03-20

**Authors:** Ragnhild Klingenberg Røed, Gunn Astrid Baugerud, Rolf Magnus Grung, Miriam Sinkerud Johnson

**Affiliations:** 1 Department of Social Work, Child Welfare and Social Policy, Faculty of Social Sciences, 60499 Oslo Metropolitan University, Oslo, Norway; 2 Department of Behavioral Science, Faculty of Health Sciences, 60499 Oslo Metropolitan University, Oslo, Norway

**Keywords:** disclosure, investigative interviewing, child abuse, child maltreatment, preschool children, interview dynamics

## Abstract

Children’s disclosure of abuse constitutes a multifaceted process i.e. critical for professionals to address promptly, ensuring the immediate protection of the child. Little is known about the patterns of disclosure among preschool-aged children. The present study investigated disclosure patterns in 131 forensic interviews with preschool-aged allegedly abused children, all of whom reported abuse during the interview. Specifically, we examined the point in the interview at which children disclosed the abuse, the types of questions asked by the interviewer prior to the disclosure, whether the children provided new information about the abuse in response to subsequent questions after disclosure, and the interviewers’ follow-up prompts following the children’s disclosure. The findings showed an average of 88.9 turns before disclosure. One-third of the children disclosed abuse during the pre-substantive phase of the interview, with almost half of these disclosing early. Even children aged 3 provided forensically relevant information across multiple turns, comparable with the 5-year-olds. However, the preschool-aged children were interviewed using techniques that were leading and involved lengthy sessions, which did not align with best practices. This may raise questions about the validity and representativeness of the findings. Implications for practice are discussed.

## Introduction

Children’s disclosure of abuse in forensic interviews is an intricate and complex process affected by a multitude of factors (e.g., Alaggia et al., 2019; Collin-Vézina et al., 2015). In the vast majority of cases, determining the truth of allegations depends substantially if not entirely on children’s recollection and disclosure of abuse (London et al., 2008). However, many children who have experienced abuse do not initially report abusive experiences at their own initiative (Lemaigre et al., 2017; McGuire & London, 2020). In fact, a recent meta-analysis found that as many as one-third of children fail to disclose abuse during forensic interviews (Azzopardi et al., 2019). Even when children have previously reported their abusive experiences to another person, which increases the likelihood of later disclosure, 7%–26% of children do not report the abuse in forensic interviews (Hershkowitz et al., 2005; Tamm et al., 2021). These patterns generally highlight the reluctance or inability of many children to disclose abusive experiences, both initially and during interviews.

Extensive research indicates that the likelihood of disclosures is affected by an intricate interplay of the children’s developmental characteristics as well as contextual and motivational factors (Gewehr et al., 2021; Lahtinen et al., 2018; Magnusson et al., 2017; Wallis & Woodworth, 2020). Of motivational factors, the emotional relationship between the child and the perpetrator, is found to inflict the disclosure process in various ways. For example, a child’s dependency on the relationship to the perpetrator and/or the perpatrator threatening to hurt someone dear to the child, or telling the child about the risk of being removed from the home if they tell, have been identified as barriers to children’s disclosure (Augusti & Myhre, 2021; Leach et al., 2017; Magnusson et al., 2017; McElvaney et al., 2014). The perpetrator has the position to frame the abuse as something special (or normal) or request secrecy, found to contribute to children’s ambivalence in communicating about the abuse due to feelings like shame, fear, or actions of secrecy (Azzopardi et al., 2019; Collin-Vézina et al., 2015; Lyon et al., 2019; Malloy et al., 2011). Of distinctive challenges faced by the youngest children, are their lack of knowledge about and access to corrective information or experiences of what constitutes appropriate (and non-appropriate) behaviour, heightening their risk of failing to understand when an act is abusive (Alaggia et al., 2019; Brennan & McElvaney, 2020; Lyon et al., 2019). These barriers are particularly related to intrafamilial abuse (Easton, 2019; Gewehr et al., 2021; Hershkowitz et al., 2006), and they become even greater when the perpetrator and the victim cohabit (Leclerc & Wortley, 2015).

The significance of gender for reporting child abuse has been studied. Some researchers have shown gender differences, with boys being more reluctant to report abuse, but gender differences in disclosure rates are not consistent (Hershkowitz et al., 2005; Lahtinen et al., 2022; London et al., 2008). A study by Lev-Wiesel and First (2018) found support for gender differences in a population-based sample, but they also found an effect of type of abuse, as victims of child sexual abuse (CSA) were more reluctant to disclose than children having experienced child physical abuse (CPA).

Children’s age has also been found to affect the probability of disclosure and informativeness (Azzopardi et al., 2019) and children under the age of 6 are less likely to intentionally talk about abuse experiences compared to school-aged children (Azzopardi et al., 2014; Pipe et al., 2004). This tendency is assumed to be related to their limited cognitive ability to recognize behaviour as abusive and, consequently, difficulties in understanding the purpose of an interview. However, because the existing findings on children’s disclosure patterns in forensic settings have largely been based on samples with large age spans, there is limited knowledge about how disclosure patterns unfold in forensic interviews with preschool children as young as 3 years old.

Several studies have reported that typically developed preschool-aged children have the ability to take part in a dialogue within the context of forensic interviews. Research finds that 3- and 4-year-olds have sufficient cognitive, verbal, and communicative skills and attentional capacity to answer questions and provide relevant information about abuse when questioned at length (Gagnon & Cyr, 2017; Hershkowitz et al., 2012). Additionally, Magnusson et al. (2017) found that preschool-aged victims were generally accurate in their reports of abuse and can describe in detail experiences directly before and after an assault (Magnusson et al., 2017). Another study found that children from the age of 3 years old could provide accurate information about a highly stressful event—removal from biological parents by Child Protective Services—both one week and three months after the event (Baugerud et al., 2014). Notably, 3- to 12-year-old children showed low consistency in information content over time due to providing a high amount of new correct information, even after three months, especially for focused questions compared to free-recall questions among the youngest. Accuracy was verified due to the researcher being present and documenting what happened during the removal (Baugerud et al., 2014).

The youngest children have cognitive capacity to recall information about past personal events, including distressing and emotional experiences. However, they employ less complex mental strategies in the formation, retainment, and retrieval of self-referent memories (Bauer, 2015; Hershkowitz et al., 2012; Pipe et al., 2004). With age, memory performance increases alongside cognitive development through such changes as improved language skills, enhanced capacity to retrieve and verbalize memories, and decreased ordinary forgetting of information (Bauer, 2015; Bauer & Larkina, 2014; Perez et al., 2022; Peterson, 2012). Overall, with increased age, the development of cognitive abilities generates more detailed, comprehensive, and consistent accounts of personal experiences (Eisen et al., 2007; Hershkowitz et al., 2012; Lamb et al., 2018).

Another aspect that plays a decisive role in the verbal dynamics in forensic interviews is the interviewer’s behavior including questioning style (Agnew & Powell, 2004; Brubacher et al., 2020; Lamb et al., 2015). Decades of research on interviewer performance have generally shown a lack of adherence to best practice guidelines (Baugerud et al., 2020; Cederborg et al., 2000; Johnson et al., 2015). Clear international consensus generally recommends the frequent use of open-ended questions in age-appropriate language and length as the preferred strategy for eliciting a narrative account (Brubacher et al., 2020; Newlin et al., 2015). Such questions encourage elaborate responses without specifying the required information (Lamb et al., 2018; Powell & Snow, 2007). Open-ended questions such as invitations tap free recall memory, and have across ages been found to provide more accurate information than questions activating recognition memory (Faller, 2007). The interviewer should stay consistently open-ended in their questioning strategy, even when asking directive, cued recall questions using previously disclosed information, such as “You said he pushed you. Tell me more about that,” and “Where did that happen?” (Brubacher et al., 2020; Hershkowitz et al., 2012; Lamb et al., 2018; Lyon, 2017). This type of directive questions guide children’s attention and scaffolds the retrieval of memories, which helps children be productive (Ahern et al., 2018; Hershkowitz et al., 2012; Kulkofsky et al., 2008).

In addition to the types of questions asked by interviewers, several other aspects of interviewer behavior may influence children’s responses in forensic interviews. One factor which risk distorting children’s accounts are cognitive biases in investigations and interviews (Ceci et al., 2016). One consequential bias is the human tendency to selectively seek, focus on and interpret information supporting one’s ownbeliefs (i.e., confirmation bias) (O’Donohue & Cirlugea, 2021). Whether a hypothesis-testing approach is applied within the interview itself can directly impact the validity of a child’s disclosure of abuse. Cognitive biases, such as confirmation bias, may inadvertently lead interviewers to focus on confirmatory evidence rather than conducting an open investigation (Ceci et al., 2016; O’Donohue & Cirlugea, 2021). Additionally, using a holistic approach, being supportive, and offering a calm, friendly, and trauma sensitive environment with interviewers and police attorneys wearing casual clothing, for example, tend to make child witnesses more resistant to inappropriate phrasing and improve accuracy (Blasbalg et al., 2020; Saywitz et al., 2017, p. 310).

Understanding more about the disclosure process among preschool-aged children is crucial. This includes exploring case characteristics that influence the timing of disclosure and young children’s capacities in a forensic interview, which can help facilitate earlier disclosures.

### The Current Study

The current field study aimed to examine the disclosure patterns in a sample of 131 preschool-aged allegedly abused children who were interviewed as part of formal criminal investigations related to allegations of CSA or CPA. All of the children in the sample disclosed sexual and/or physical abuse during the initial forensic interview. Specifically, we examine the children’s disclosure patterns by identifying *when* during the interview they initially disclosed abuse and how many turns the children remained on topic, providing forensically relevant information after the initial disclosure. The unit of measuring, “turn”, refers to a pair of question–response interaction between the forensic interviewer and the child witness. Moreover, we examined how interviewers facilitated children’s disclosure of abuse by identifying the types of prompts used prior to disclosure and as follow-up prompts after disclosure. The following research questions were addressed:

(1) At what point during the forensic interview did the children disclose abuse?
(2) What types of interviewer prompts were asked prior to the initial disclosure of abuse?
(3) During how many turns did the children provide forensically relevant information after their initial disclosure?
(4) What type of follow-up prompts did interviewers use to immediately facilitate the child’s initial disclosure?

## Methods

### Sample and Case Characteristics

The sample consisted of 131 real-life forensic interviews of preschool children aged three to six years old (*M*_*age*_ = 57.6 months, *SD* = 9.2, range 37–78 months), of whom 84 (64.1%) were girls and 47 (35.9%) were boys. The frequency of the different age groups was as follows: 3-year-olds (*n* = 19, age range 37–47 months), 4-year-olds (*n* = 57, age range 48–59 months), 5-year-olds (*n* = 47, age range 60–71 months), and 6-year-olds (*n* = 8, age range 72–78 months). None of the children had attended school at the time of the interview; therefore, the sample was labeled preschool-aged children. Most of the children (81%, *n* = 106) were interviewed by a female interviewer and 19% (*n* = 25) were interviewed by a male interviewer.

The interviewees were alleged victims of CSA (*n* = 86, 65.6%) or CPA (*n* = 45, 34.4%). The alleged incidents of abuse ranged from exposure or fondling of the perpetrator’s genitals to penetration and from spanking to beating. All cases were classified by the prosecutor as either a CSA or a CPA case due to the primary criminal provision. The sample included cases of suspected intra-familial abuse (90.1%), of which 66.1% were CSA and 33.9% were CPA, and extra-familial abuse (9.9%), of which 61.5% were CSA and 38.5% were CPA. All of the children in the sample made specific disclosures of sexual and/or physical abuse during the forensic interview, including event-specific and person-related information regarding abusive incidents. Of 131 preschool-aged witnesses, 30% (*n* = 39) disclosed abuse in the pre-substantive phase and 70% disclosed abuse (*n* = 92) in the substantive phase.

The sample in this study was selectively chosen from a larger nationwide sample of preschool-aged children. The selection of cases was based on the children’s disclosure of abuse during their first forensic interview with the police. This research was part of a larger study funded by the Norwegian Ministry of Justice and Public Security, where the main aim was to assess the quality of forensic interviews in a national sample of preschool-aged children. The forensic interviews were conducted as part of formal criminal investigations of the cases, all of which progressed to prosecution. There was no information in any cases regarding the existence of supporting evidence of whether the disclosed abuse had occurred or were non-credible disclosures (false positives). Non-credible disclosures can result from prior undue influence by a third party with hidden motives or due to improper questioning techniques by the recipient of pre-interview disclosure or by the forensic interviewer (Azzopardi et al., 2019). The rate of false accusations of CSA has been estimated and found to occur at non-negligible rates of two to five percent (O’Donohue et al., 2018). The likelihood of the allegations being false positives cannot be rejected, however, it was deemed by the prosecution to be sufficiently certain that criminal acts had occurred, leading to charges being filed in all cases in the sample.

Ethics approval was granted by the State Attorney, the National Police Directorate, the Data Inspectorate, and the university’s Data Protection Officer.

### The Nordic Barnahus Model

All interviews were conducted at a Norwegian *Barnahus* (equivalent to a Child Advocacy Center) located within one of the country’s 12 police regions. There are 11 *Barnahus,* all situated within the justice and police sectors. In brief, *Barnahus* is a model for interdisciplinary and co-located work in police-reported cases involving children. The aim is to ensure a multi-professional approach in a child-friendly environment through collaboration among law enforcement, child protective services, pediatricians, dentists, and mental health services (Johansson et al., 2017). Forensic interviews are regarded as court hearings, that is, the interviews are video recorded at the *Barnahus* and played in court, being regarded as valid witness statements. Forensic interviews at the *Barnahus* are led by a trained police attorney obliged to ensure the suspect’s right to cross-examine the child. The suspect’s attorney is present at the *Barnahus* and provides questions for the specially trained forensic interviewer to ask the child. Children are thereby protected from having to testify in court and meet for cross-examinations within the criminal justice system (The Norwegian Criminal Procedure Act (1981, §239; Regulation of Facilitated Interviews, 2015). Attorney(s) for the witness (es) and, when relevant, the accused party are present at the *Barnahus* and watch the interview in real time with police investigators and *Barnahus* staff providing supervision (Johansson et al., 2017).

### Interviewers and Interview Methods

The specially trained interviewers were police officers with a bachelor’s degree in general policing from the Police University College addressing tactical investigation including principles of hypotheses-driven investigation and training in structured adult interviewing (Norwegian Police University, 2024). In addition, they had completed a two-step part-time continuing education program to specialize in forensic interviews of children and other vulnerable groups (Baugerud et al., 2020; Norwegian Poilice University College, 2021; Norwegian Police University, 2022). To apply for child interview training, applicants must meet the requirements in general interview training, have work experience within criminal investigation, and have completed forensic interviews with adults. The police interviewers attended a one-year training program on the regular interview model for children aged 6–16 years, called the Dialogical Interviewing Method (DCM) (Langballe & Davik, 2017, p. 165). This is a phase-structured model building on internationally recommended empirically based guidelines for interviewing children, such as the NICHD protocol (Lamb et al., 2007; Orbach et al., 2000; Sternberg et al., 1996). After DCM training, the police interviewers had to have conducted at least 30 child forensic interviews over the past three years before they applied for training in the interviewing method for preschool children and other vulnerable witnesses, referred to as the Sequential Interview (*SI*) model (Langballe & Davik, 2017, p. 165). Each of the continuing education programs (i.e., the DCM and SI-model training) was estimated to require 420 hours (15 ECTS) and was commonly finished within one year, including a final exam. The training programs were built around 105 hours and 90 hours of group sessions, respectively, and lectures on subjects such as communication, developmental and witness psychology and interdisciplinary cooperation. In addition, trainees conducted a limited number of investigative interviews at *Barnahus* under the supervision of a senior police investigator. The police trainees wrote evaluative logs and received individualized feedback when meeting at the University College (Myklebust, 2017; Norwegian Poilice University College, 2021; Norwegian Police University, 2022).

Based on the Extended Forensic Interview model (Carnes et al., 1999, 2001) the SI-model has been adapted to the needs of 3- to 6-year-old children by being structured in multiple sequences (Langballe & Davik, 2017). The overall structure is phase-based, just like DCM, but the substantive phase is split into sequences with breaks between. This allows age-appropriate lengths for the interview sequences and more extensive interdisciplinary cooperation in the breaks (Langballe & Davik, 2017, p. 165). In advance, an extended preparatory phase for gathering information about the child (i.e., intellectual and social functioning, context) and conducting an interdisciplinary consulting meeting is included as part of the SI-model. In the consulting meeting with the police attorney, the interviewer, Barnahus counselor, the child’s counsel, and if relevant, the child welfare service, services discuss information from prior investigative activity and knowledge about the child’s context and level of functioning relevant for the day of the child interview and the child’s security.

The SI-model starts with sequence 1, the pre-substantive phase, which focuses on rapport building, establishing the dialogue, and rules of communication. After a break, in sequence 2, the substantive phase, the interviewer directs the child’s attention to the topic of alleged abuse with the aim of eliciting the child’s free narrative account before further probing. Next, follow-up questions like cued invitations and directive questions are employed (e.g., “You told me… Tell me more about…?”). The substantive phase can last for multiple sequences with short breaks between them, followed by the last sequence, the closure phase. There is no available published protocol for the SI model (Langballe & Davik, 2017).

### Procedures

The interview transcripts were word-for-word transcribed dialogues of the forensic interviews, from the first to the last turns in the phase of the interview in which forensically relevant information about the alleged abuse was reported by the child witness. Specifically, this means that transcriptions comprised dialogues from both the pre-substantive phase and the substantive phase of the interview in accordance with different practices in the police districts. In the current sample, 39 (30%) of the interviews were transcribed from the first turn in the pre-substantive phase (Sequence 1), while the rest of the sample, comprising 89 (70%) interviews, was transcribed from the substantive phase. All personal information was removed from the transcripts before coding to ensure anonymity and confidentiality. The interview transcripts were manually coded by human coders using a detailed and exhaustive coding scheme that captured the verbal dynamics between the interviewer and the child interviewee.

Interviewers’ questions and utterances were coded using the NICHD investigative interview coding scheme (see Lamb, 1996; Lamb et al., 2007), used in earlier studies of forensic interviews following the same interviewing guidelines (Baugerud et al., 2020, 2023, Johnson et al., 2015, 2025). The coding procedure differentiated between recall prompts (invitations, cued invitations, or directive [wh-] prompts), recognition prompts (option-posing [yes/no and forced choice or suggestive prompts]), and facilitators. *Invitations* referred to prompts asking the child to talk freely about the event (e.g., “Tell me what you have come to talk about.” “Tell me everything.”). *Cued invitations* included asking for more information about a situation or person/object already mentioned by the child (e.g., “Tell me more about the night your aunt came.”). *Directive wh-questions* referred to prompts asking for clarification or requesting specific information about a theme already mentioned (e.g., “What did your brother say?” “Where was your mother?”). *Option-posing questions* referred to yes/no prompts asking the child to affirm or deny something that was stated (e.g., “Was your brother with you all the time?”) or forced-choice prompts asking the child to choose between two or more optional answers (e.g., “Did you have your clothes on or take them off?”). *Suggestive prompts* were coded when requesting a certain response or introducing information not mentioned previously (e.g., “And then he hit you, didn’t he?”, when the child has not previously mentioned that anyone had hit him/her). *Facilitators* were coded when statements summarized or paraphrased the child’s previous statements or motivated further accounts (e.g., “I see” or “M-hmm”).

The children’s response behaviors in the forensic interviews were coded into two main categories—productive or nonproductive responses—according to their relevance to the alleged event. *Productive responses* included those in which the children confirmed (“yes”) or denied (“no”) and those in which children provided new forensically relevant information about the allegation, including details identifying or describing individuals, objects, events, or actions related to the investigated incident(s). *Non-productive responses* included “I don’t remember,” “I don’t understand,” off-topic responses, restatements of previous utterances (i.e., repeating information), and non-response (silence).

To examine disclosure patterns, the current study extracted sequences of each interview in which the child’s initial disclosure was elicited as a response to an interviewer prompt and the follow-up interviewer prompt was associated with the child’s productive response. The initial disclosure was operationalized as the incident in the interview situation in which the child reported forensically relevant information regarding the alleged event, including person- and situation-specific information, the first time (Lahtinen, 2022). One separate code was set for the initial disclosure in addition to being coded as a productive response. The codes for the interviewers’ prompts and for the children’s responses were mutually exclusive (i.e., only one code could be given for a particular speech act) and exhaustive (i.e., there was always a code).

### Inter-rater Reliability Between Coders

Inter-rater reliability for the presence of disclosure, interviewer prompts and children’s responses was calculated. Two native speakers who were blind to the study’s purpose were trained in the coding of interviewers’ prompts and children’s responses by an experienced coder (last author) on an independent set of interview transcripts until they reached 90% agreement. Then, 30% (*n* = 40) of the transcripts were independently coded by the coders to ensure that they remained equivalently reliable. The remaining interviews were split in half between the two coders. The raters achieved 100% agreement regarding the coding of the children’s disclosure (present vs. not present). Excellent intra-rater reliability was achieved for both interviewers’ prompts (Cohen’s κ = 0.89) and children’s responses (Cohen’s κ = 0.91).

### Analyses

All data were analyzed using SPSS Version 28. Preliminary analysis included descriptive statistics of the sample, including the children’s age, gender, and turn of disclosure. In the analyses of prompts asked just before and as follow-up to the children’s initial disclosures, ‘invitations’, ‘cued invitations’, ‘directive (wh-)' questions, and ‘facilitators’ were considered as individual prompt categories. Prompts coded as “yes/no” and “forced choice” were collapsed into the category “option-posing” questions. Leading, repeating, and referring prompts were collapsed into the category “suggestive”.

Descriptive analyses were conducted to identify in which turn the disclosure initially happened during the interview and in which phase of the interview, the pre-substantive phase or the substantive phase. The turn in which a disclosure occurred ranged from 1–288 (*M* = 88.9, *SD* = 73.0) independent of phase, making it functional to split turns into intervals as follows: 1–20 (early disclosure), 21–50 and 51–100 (mid-section), and 101–150 and above 151 (late disclosure). Furthermore, independent sample t-tests were carried out with age and gender as independent variables, and turns for initial disclosure as well as total length of the forensic interview regarding number of turns were dependent variables. A two-way ANOVA was conducted to compare interview length across age groups while accounting for the phase in which disclosure occured.

When examining the type of prompts asked prior to the initial disclosure and as follow-ups, descriptive analyses including cross-tabulations with Pearson chi-square tests, were conducted. To explore the degree to which preschool-aged children 3–6 years old continued to disclose new productive information in conversational turns after initial disclosure, a Kruskal-Wallis test with Bonferroni correction was conducted. Mann–Whitney U tests were performed to compare whether there were differences in the amount of information children provided (i.e., productivity) related to whether they were subjected to CSA or CPA, and whether the abuse occurred within or outside the family.

## Results

### Interview Length and Distribution of Interviewer Questions

Among the 131 forensic interviews, data was missing on the duration of the pre-substantive phase in cases where children disclosed abuse during the substantive phase. For that reason, the sample was explored as two sub-samples according to interview phase. Among the 39 interviews with disclosure in the pre-substantive phase, the shortest interview contained 73 turns and the longest had 543 turns. The average length was 248 turns (*SD* = 108.9). Further, a two-way ANOVA on the length of the interview grouping participants by age, found that the average number of turns in interviews of 3–4-year-olds (*n* = 26) disclosing in the pre-substantive phase was 246.7 (*SD* = 111, range 101–543). Among 5–6-year-olds (*n* = 13) disclosing in the pre-substantive phase, the average length of the interview was 251.2 (*SD* = 109, range 73–433). There was no significant difference in length between the age-groups.

In interviews where the preschool-aged children reported abuse during the substantive phase (*n* = 92), the length of the interviews ranged from 88 to 432 turns (*M* = 217, *SD* = 85.2), bearing in mind an unknown length of the pre-substantive phase. A two-way ANOVA revealed that children aged 3–4-year-old (*n* = 50) were, on average, asked 211.9 prompts (*SD* = 83.5, range 88–411). Children aged 5–6-year-old (*n* = 42) were asked, on average, 222.9 prompts (*SD* = 87.7, range 98–432). There was no significant difference in length of the interviews between the age-groups.

### Disclosure Patterns Among Child Interviewees

When reporting abuse in the pre-substantive phase, the initial disclosure ranged from the first turn to turn 182. More precisely, it took on average 44 turns before disclosure (*SD* = 45.8). When children disclosed abuse during the substantive phase, the initial disclosure ranged from the first turn to turn 288, with an average of 108 turns, *SD* = 74.2.

### The Turn of Initial Disclosure

**Figure 1.**
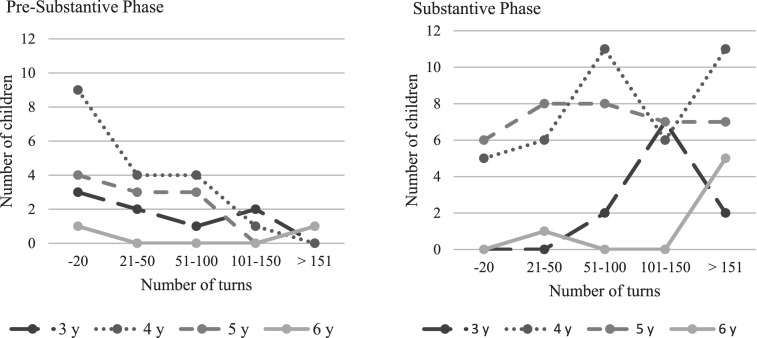
Turns of initial disclosure.

To explore the exact place in the interviews when the children initially disclosed abuse, the sample was separated by phase according to the initial disclosure (Figure 1). Conducting cross-tabulations with age per year and turn (interval) at initial disclosure in the pre-substantive phase, revealed that of 39 children, 43.6% (*n* = 17) children disclosed within 20 turns, of whom half were 4-year-olds. It took 159 turns at the most in this sub-sample. As many as 12.8% (*n* = 5) of the children reported after more than 100 turns.

Analyses of the 92 child interviews where children disclosed abuse during the substantive phase revealed that only the 4- and 5-year-olds (12.0%, *n* = 11) disclosed abuse within the first 20 turns. For almost half of the children, 48.9% (*n* = 45), it took 100 or more turns until they disclosed abuse-related information, one being interviewed with 288 turns before initial disclosure.

Previous research has shown that children’s age and gender as well as type of abuse (CSA vs. CPA) and children’s relationship to the suspect often predict children’s disclosure. To assess whether these variables affected the disclosure process in the forensic interviews, independent sample t-tests were conducted. The analysis revealed that neither boys and girls nor children in different age groups differed significantly on the timing of initial disclosures in the interviews. Regarding the type of abuse disclosed and the type of relation to the suspect, no differences were statistically significant.

### Last Question Posed Before Initial Disclosure

**Figure 2.**
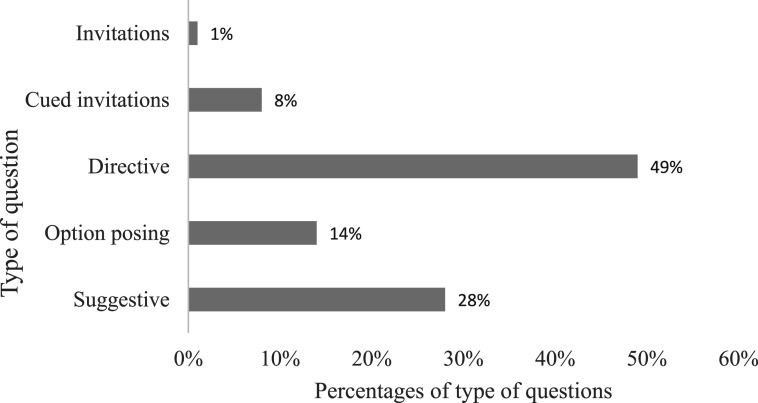
Percentages of question types immediately preceding initial disclosure of abuse.

Investigating the type of last question posed before the children’s initial disclosure of abuse revealed that almost half of the interviewers asked directive (wh-) questions (49%) across age and independent of phase of disclosure. Figure 2 depicts the distribution of question types. Leading questions immediately preceded the initial disclosure in 42% of the cases of which 28% were suggestive prompts and 14% were option-posing questions. Related to initial disclosure, only one invitation was posed and that was the 110^th^ prompt asked. Only 8% of the questions that immediately preceded the initial disclosure were open-ended and focused.

**Figure 3.**
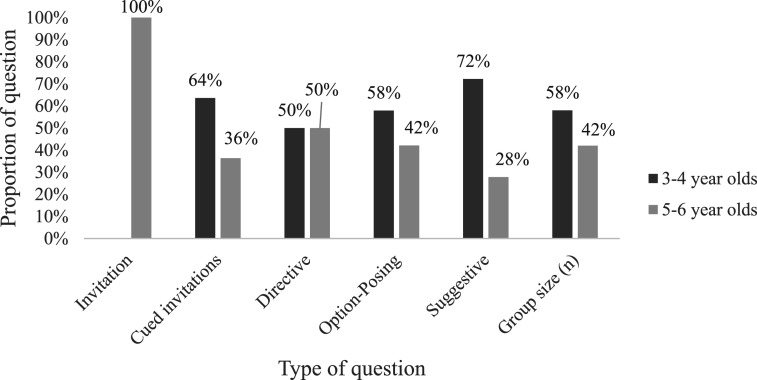
Distribution of question types immediately preceding initial disclosure by age.

Breaking the sample into 3–4-year-olds (*n* = 76, 58%) and 5–6-year-olds (*n* = 55, 42%) independent of phase of the interview, the analysis revealed that the youngest children were asked proportionally more suggestive prompts and cued invitations, and fewer directive questions than the oldest children (Figure 3). The proportional distribution of option-posing questions mirrored the size of the age groups, at 58% and 42%, respectively, with these questions being evenly distributed across both age groups. The analyses revealed no significant association between the groups and the distribution of question types (*p* > .05).

### Number of Turns During which the Child Provided Forensically Relevant Information

A cross-tabulation was conducted to investigate the extent to which preschool-aged children stayed on the topic of abuse after their initial disclosure response. The results revealed that the number of turns varied from 1 to 12 (*M* = 4, *SD* = 2.5) for continuous reporting of new and detailed information related to the alleged event. Of the 131 preschoolers, 25 provided forensically relevant information for 6 to 9 turns, while 5 children continued for 10 to 12 turns. The majority–58 preschoolers–remain on topic for 3 to 5 turns before changing the subject.

**Figure 4.**
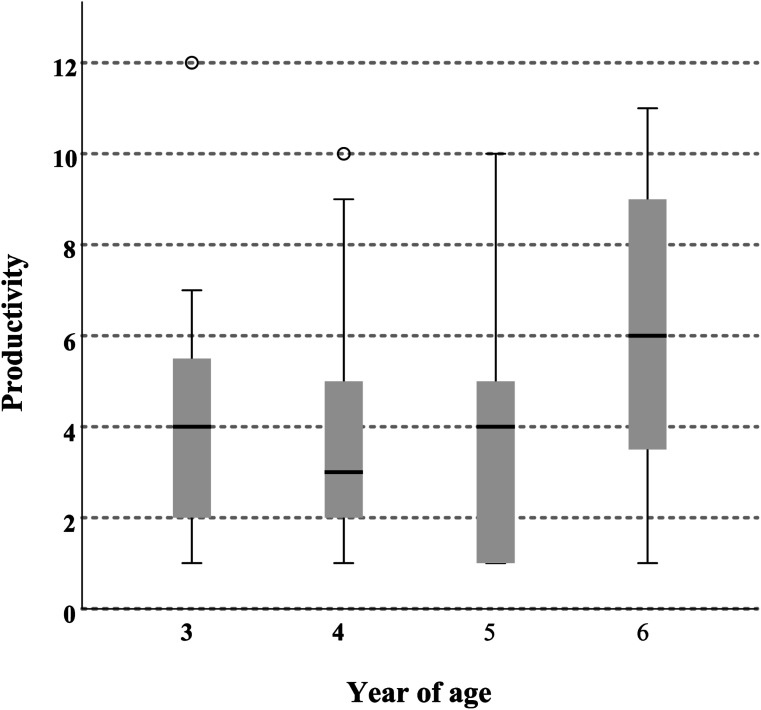
Number of continuous productive responses after initial disclosure sorted by age.

To examine the relationship between children’s age and their continued disclosure of abuse-related information, the sample was studied separately for each year of age. A Kruskal–Wallis test was conducted with Bonferroni correction for multiple comparisons. The results revealed a statistically significant difference in the mean productivity between children 4 and 6 years old (*p* = .04), where the oldest stayed on topic for significantly more turns. As depicted in the boxplot in Figure 4, the mean score for 3- and 5-year-old children was the same, with an average of four turns of providing new information related to the alleged abuse. Furthermore, two extreme values were marked with one 3-year-old child who continued for 12 turns and one 4-year-old child who continued for 10 turns.

To explore the process of disclosing abuse related to abuse type, an independent-samples Mann–Whitney U test was performed to compare the two types of abuse: CSA (*n* = 86) and CPA (*n* = 45). The results revealed no significant difference when comparing the rank means of the number of turns during which the children provided information (CSA rank mean = 66.1, CPA rank mean = 65.7). The number of children’s information-providing turns did not differ by whether they experienced intrafamilial or extrafamilial abuse.

### Type of Prompt Used to Follow up the Initial Disclosure

**Figure 5.**
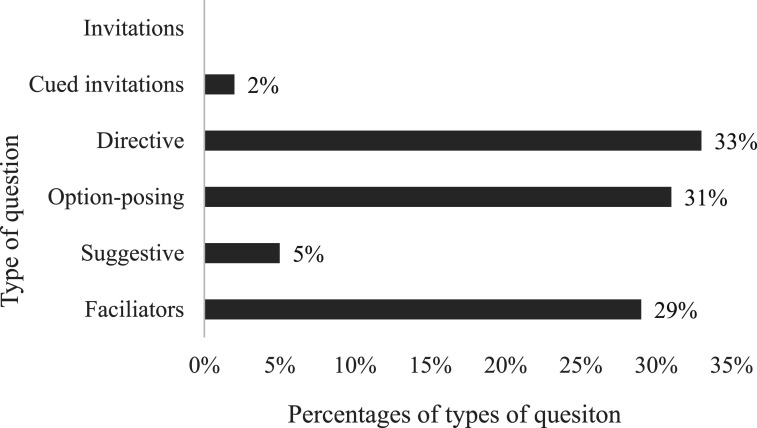
Percentages of types of prompts interviewers asked as follow-ups to disclosure.

The assessment of how the interviewers followed up on the initial disclosure to facilitate elaboration revealed three main tendencies related to the type of prompts chosen. Interviewers asked directive (wh-) questions (33%), option-posing questions (31%), or they used facilitators (29%). Option-posing prompts were mainly yes/no questions (28%). Cued invitations were used in 2% of the interviews, and no invitations (Figure 5).

When the sample was divided into groups of 3–4-year-olds and 5–6-year-olds, the analysis revealed that directive and option-posing prompts, as well as facilitators, were proportionally evenly distributed. However, the youngest children were asked proportionally more suggestive prompts and fewer cued invitations than the oldest children, but the percentages of recommended prompts were low (5% and 2%, respectively).

## Discussion

This study indicates that preschool-aged children as young as 3–4 years old have the capacity to provide forensically relevant information about investigated incident(s), results consistent with previous studies (Gagnon & Cyr, 2017; Hershkowitz et al., 2012). The children demonstrated perseverance in the interview setting by remaining engaged over numerous conversational turns, regardless of the phase in which they initially disclosed the abuse. The average length of the interview was 248 turns (range 73–543) among children disclosing in the pre-substantive phase. Nearly one-third of the children reported abuse during the pre-substantive phase, with 17 children out of 131 providing abuse-related information within the first 20 turns. Disclosure within the first 20 turns may indicate that preschoolers understand the context of the interview, offering a nuanced perspective on previous research that suggests children under 6 years old are less likely than older children to recognize acts as abusive and, consequently, grasp the interview context (Azzopardi et al., 2019; Leach et al., 2017; Pipe et al., 2004).

The length of the interviews varied, with the majority lasting a substantial number of conversational turns. Considering child developmental factors, one could expect longer interviews with an increased age, but no significant difference was found across age. The interview lengths, up to 543 turns in total in the pre-substantive phase, cause concern due to the young children’s limited attentional and cognitive capacity. Their limited capacities raise a concern about children responding to words or phrases based on recognition and not understanding (Baugerud et al., 2020; Lamb et al., 2015; Lyon et al., 2019). This can compromise the accuracy of their accounts and may lead to false allegations, especially when interviews are conducted using poor questioning techniques. Of the preschool-aged children disclosing in the substantive phase, almost half made their initial disclosure after being asked more than 100 questions/utterances in addition to the unknown number of questions being posed in the pre-substantive phase. All interviews were conducted in one day.

Previous research has found that the youngest child witnesses respond with fewer details to open-ended questions. Nevertheless, invitations and cued invitations are recommended across all ages (Lamb et al., 2018; Lyon et al., 2019) to elicit more detailed and accurate information than directive, option-posing, and suggestive questions (Brown et al., 2013; Hershkowitz et al., 2021). The current study showed that interviewers relied heavily on directive (wh-) questions (49%) prior to the children’s initial disclosures across ages and independent of when the child reported abuse during the interview. Additionally, leading questions were the most applied, with one-third of the children being asked a suggestive question and 14% an option-posing question immediately prior to the initial disclosure, while only 8% of the children were prompted with a cued invitation. The preferred broad invitation was used once (in turn 110). A low percentage of open-ended questions and a high percentage of suggestive questions are problematic, particularly when interviewing the youngest children, due to the association between suggestive questioning and an increased risk of erroneous responses, which may lead to false reports (Kulkofsky & London, 2010, p. 217; Saywitz et al., 2017, p. 310). Moreover, we also found a tendency towards interviewers posing suggestive questions to the youngest as the 3–4-year-olds were asked proportionally more suggestive questions and fewer directive questions than the 5–6-year-olds, increasing concerns about the quality of the questioning. No known study has focused on the type of question that elicited initial disclosure, but evaluative studies of forensic interviews with preschool-aged children have found similar results, with a lack of open-ended questions and high proportions of directive (wh-) and suggestive questions (Baugerud et al., 2020; Cederborg et al., 2000).

Furthermore, we found, regardless of validity, that two-thirds of the sample provided forensically relevant information across three or more turns, with a maximum of 12 turns, although preschoolers are generally believed to give less extensive accounts (Hershkowitz et al., 2012; Lamb et al., 2003). This finding may align with previous research that has found 3 to 4-year-olds have the capacity to provide forensically relevant accounts, also across multiple turns (Baugerud et al., 2014; Hershkowitz et al., 2012). Interestingly, we even found that 3-year-olds showed the same capacity to stay on topic as the 5-year-olds. These findings add to knowledge about young children’s narrative style, characterized by reporting personal experiences piece by piece through an ongoing dialogue (Brubacher et al., 2020). Connecting findings of narrative style (productivity) with the type of question eliciting disclosure, illustrates the challenging task of child interviewing. Both being asked suggestive questions and being asked an extensive number of questions before initial disclosure at this young age raises worry of contamination if suggestive questions precede initial disclosure or if the child is tired out from being asked a high number of questions.

Interviewing children who report abuse-related information is a rich opportunity for open-ended questioning, but the current study revealed that more than one-third of the interviewers chose option-posing, mainly yes/no-questions, or leading questions as the first prompt after initial disclosure. Such non-recommended questioning paired with the child providing information about an alleged event, involve risk related to dynamics such as a yes-tendency among the youngest witnesses and the tendency to please the interviewer independently of the accuracy of the information provided (Powell & Snow, 2007; Stolzenberg & Lyon, 2014). Further, relatively few suggestive questions were posed following up on disclosure, but each one of them raises great concern about high error rates related to the non-recommended questions used prior to disclosure.

In this study, preschool-aged children were found to report information about abuse in a forensic context and do that across multiple interviewer questions. All child interviews were part of child abuse cases that proceeded to court. However, the validity of the disclosures must be questioned. The interviews were extensively lengthy and the type of questions used both before and as follow-up to disclosure gives rise to great concern. The police interviewers were all specially trained and the interviews were conducted at Barnahus, making it timely to direct the attention towards training and maintenance of interviewing skills.

### Strengths and Limitations

The current study has several strengths. Firstly, the sample includes a considerable number of real-life investigative interviews with preschool-aged children of which the majority were 3–4 year olds allowing for analysis with both 3- and 4-year-olds as separate groups. Furthermore, the coding of conversational turns revealed the nature of the interviewers’ questioning preceding initial disclosure and immediately following disclosure and identified where in the interview preschool-aged children disclosed abuse information initially. However, there are several limitations to be acknowledged.

Dividing the sample into two groups based on age, 3–4- and 5–6-year-olds, gave a difference in sample size (*n*) of 76 versus 55 children, respectively. For analyses of the proportional distribution of type of question by age group (Figure 3) this was an important consideration. Further, when exploring productivity, we found 4- and 6-year-olds to be different in amount of information provided with the oldest reporting the most. The sample sizes differed, with eight children in the 6-year-old group and 57 children in the 4-year-old group. The sample size can be considered a limitation, as is often the case in field studies, where large datasets are desirable. Further limitations include the many unexplored factors related to young children’s disclosure of alleged abuse. One such factor is the unknown effect of the quality of rapport-building found to impact the disclosure process, the missing data on the actual time the interviews ran, and whether the child had disclosed abuse-related information prior to the interview (Hershkowitz et al., 2006; Hershkowitz & Lamb, 2020).

### Implications and Future Research Directions

The current study aimed to explore and understand patterns of disclosure among preschool-aged children and their capacity to provide relevant information in an interview context. Findings indicate that child interviewing is a challenging task, as trained interviewers did not consistently adhere to best-practice guidelines. This study highlights the importance of enhancing interview training through the development of structured training programs, the integration of innovative approaches, and the implementation of strategies to maintain interviewing skills (Baugerud et al., 2024; Korkman et al., 2024). Key elements identified pre- and post-training include practical skills training with feedback, individualized feedback, and peer review within a structured evalution framework to ensure continuous improvement and adherence to best practice recommendations.
